# The influence of widowhood and social engagement on cognitive impairment among Chinese older adults and factors mediating their association

**DOI:** 10.7189/jogh.14.04193

**Published:** 2024-09-20

**Authors:** Mingyuan Sheng, Kathleen Young, Ying Li, Yeyuan Zhang, Jiale Wang, Shuhan Jiang

**Affiliations:** 1 School of Humanities and Management, Zhejiang Chinese Medical University, Hangzhou, China; 2 School of Public Health, Zhejiang Chinese Medical University, Hangzhou, China; 3 Department of Health Sciences, MPH and Public Health Education Programs, California State University, Northridge, California, USA; 4 School of Public Health, Xi'an Medical University, Xi’an, China; 5 School of Nursing, Shaoxing University Yuanpei College, Shaoxing, China

## Abstract

**Background:**

Prior studies exploring the impact of widowhood on cognitive impairment in later life have been focussed on the USA and Europe. We aimed to explore the mediating role of social engagement, health behaviours, and subjective well-being in the association between widowhood and cognitive impairment in the Chinese population.

**Methods:**

We conducted a study on 7796 older individuals enrolled in the 2018 wave of the Chinese Longitudinal Health Longevity Study. We used logistic regression models to analyse the impact of widowhood on cognitive health among older adults and performed mediation analysis to determine possible mediating factors in this relationship.

**Results:**

Widows and widowers had a higher risk of having cognitive impairment than married older adults (95% confidence interval (CI) = 1.312, 2.279). The results from structural equation modelling (SEM) provided a good fit to the observed data (χ^2^ = 24.909; *P* = 0.00) and indicated that the effect of widowhood on cognitive impairment was partially mediated by social engagement, lifestyle behaviours, and subjective well-being (β = 0.075; *P* < 0.01).

**Conclusions:**

Our findings contribute to existing research on the mechanisms underlying the association between widowhood and cognitive impairment among older individuals, suggesting a need for policies targeted at the specific needs of this vulnerable population, such as the maintenance of social interactions, adoption of a healthy lifestyle, improvement of subjective well-being, and provision of necessary support systems.

Population ageing has been a growing concern for public health globally. This is especially true for China, whose population is ageing much faster than previously reported, with projections suggesting it will have more than 400 million residents who are ≥60 years of age by 2040 [1]. This would also lead to the prevalence, morbidity, and mortality of several diseases increasing sooner than expected, particularly for chronic non-infectious diseases, placing a burden on society and families to allocate resources for medical treatment, therapy, and health care for seniors.

To exacerbate these burdens further, approximately one-quarter (18.95%) of Chinese older adults had previously reported suffering from cognitive impairment [2]. As people with cognitive impairment frequently struggle with daily chores and require extensive medical and personal care, maintaining cognitive function remains a key part of health and well-being in later life [3–5]. Prior research has found a variety of diseases and bad lifestyle habits to be closely associated with the occurrence and development of cognitive impairment, with hypertension and diabetes, poor social relationships, alcohol consumption, a lack of formal education, a lack of physical exercise, and sleep disorders emerging as important and preventable risk factors [6,7].

The number of widowed older adults is predicted to increase to 118.4 million by 2050 due to gender-based disparities in life expectancy and China's ageing population [8]. Although there are several studies on the impact of widowhood and cognitive impairment on this population, most have been conducted in the USA and Europe and have treated marital status exclusively as a control variable. Existing studies on the Chinese population, in turn, have not yet focussed on how lifestyle changes after widowhood affect this relationship [9]. Several factors can influence the development of cognitive impairment [10]; however, some can be avoided through changes in an individual’s lifestyle, even in later life [11].

Several studies have indicated the health benefits of marriage, as it ensures social, psychological, and financial resources while also encouraging people to maintain healthy lifestyles [12]. For example, spouses can motivate and support each other to quit smoking, engage in physical activities, or maintain a healthy diet, thereby potentially reducing the risk of death and dementia health [6,13]. However, for older adults, widowhood following the death of a partner emerges as an especially traumatic and challenging life experience [14–16]. Research has shown that the lifestyles of widowed older adults significantly differ from those of non-widowed adults. For example, widows and widowers were found to be at higher risk of poor nutrient intake, declined sleep quality, increased alcohol consumption, and initiating or restarting smoking following their partner’s death [15,16]. Older adults simultaneously experience a decline in social engagement, as family members and friends who had been reliant on the recently deceased spouse to maintain close relationships gradually move away [17]. They are also known to develop negative emotions such as depression, anxiety, and loneliness following a period of grieving [18], with evidence suggesting an association between widowhood and poor mental health [19,20]. The mental health of older widows and widowers directly affects their attitude toward life satisfaction and well-being [21]. Yet despite a consensus that mental health is essential for active healthy ageing, few studies have explored the role of subjective well-being in the relationship between widowhood and cognitive impairment.

Research comparing widowed and non-widowed older individuals found that widowhood was associated with a higher risk of cognitive impairment. Stress and grief following the death of a spouse were also found to increase a widowed individual’s risk of depression and other chronic health problems, as well as dementia and cognitive impairment [22,23]. A cohort study with a 17-year follow-up involving 6766 older adults in the USA discovered a linear connection between widowhood duration and cognitive decline [24], while a longitudinal study with an eight-year follow-up in Korea showed that widowed older adults experienced considerable cognitive deterioration compared to non-widowed individuals [25]. Although the link between widowhood and cognitive function in Chinese older adults has received less attention, a study in four cities reported similar results [26].

The mechanism between widowhood and cognitive function is frequently discussed through the stress and marital resource models [27]. According to the stress model, hippocampal atrophy may result from emotional trauma-fueled stress-induced glucocorticoid secretion [28]. The marital resource model, in turn, proposes that marriage helps one establish or obtain social interaction, social support, and financial resources, which are associated with better cognitive function in ageing [28]. Marital interaction can also provide daily cognitive stimulation for the older adult, which is crucial for maintaining cognitive function [29]. Furthermore, emerging biomarker research discovered independent and interactive relationships among widowhood, β-amyloid levels (an abnormal brain protein associated with Alzheimer disease), and cognitive decline [30]. These mechanisms suggest that widowed individuals are at a higher risk of cognitive impairment than married ones.

Aside from the loss of economic or social support, widowed individuals may also experience spiritual loss, loneliness, and other inconveniences in daily life, resulting in further serious health consequences. A study among community-dwelling older Chinese adults in Singapore found that widowhood was associated with a higher risk of cognitive impairment than being married, but the link was statistically significant for men only [31]. However, in their population-based research on 4370 older adults, Shahar et al. [32] found no correlation between widowhood and dementia. Another study conducted in three Chinese communities found no significant correlation between being widowed and cognitive scores after controlling for covariates [27]. This may be partially attributed to the fact that widowhood has variable effects depending on social and cultural contexts and that the importance of various pathways relating widowhood to health may vary by gender and regional norms [14]. The relationship, therefore, requires further studies.

Previous research in developed countries has established a positive association between social engagement and improved health outcomes across multiple domains, including mortality, morbidities, well-being, cognitive function, and life quality [33,34]. For example, individuals with higher levels of social activity were found to typically have greater cognitive functions than those who are less socially engaged [35]. This might be due to the emotional, behavioural, and physiological benefits of social interaction on the functional abilities of older individuals [36], as older adults who are highly socially engaged are less likely to experience functional disability due to the maintenance of their cognitive ability [37]. Additionally, engaging in protective health behaviours such as an active lifestyle can improve the underlying relationship between social engagement and health [38].

With these research gaps in mind, we designed a study based on a nationwide sample of older Chinese individuals to explore the potential mediating roles of social engagement, health behaviours, and subjective well-being in the association between cognitive impairment and widowhood. We hypothesised that widowhood directly impacts both cognitive health and social engagement, and that it indirectly influences cognitive health through its effects on post-widowhood social engagement, subjective well-being, and various health-related behaviours, including sleep duration, physical activity, alcohol consumption, smoking, and dietary habits.

## METHODS

### Data source and study population

We retrieved data from the 2018 wave of the Chinese Longitudinal Healthy Longevity Survey (CLHLS). The CLHLS is the largest collection of survey data on the older adult population in China, encompassing 23 provinces inhabited by approximately 85% of the Chinese population. Following a baseline survey using a self-administered questionnaire in 1998, additional waves were conducted in 2000, 2002, 2005, 2008, 2011, 2014, and 2018 to investigate the health status and associated social, behavioural, and biological aspects among Chinese older adults recruited on a volunteer participatory basis. After we screened for invalid values and outliers, we selected 3605 men and 4191 women within the 2018 survey, comprising 1367 widowed men and 2767 widowed women (**Table 1**).

**Table1:** Table of variable assignments

	Measurement
**Dependent variable**	
Social engagement	1 = impaired social engagement; 0 = healthy social engagement
Cognitive health	1 = impaired cognitive; 0 = no cognitive impairment
**Independent variables**	
Widowhood situation	1 = widowed; 0 = married and living with a spouse
**Intervening variable**	
State of mind	
*Subject well-being*	Scores ranging from 5 to 25
Health behaviour	
*Dietary behaviours*	Scores ranging from 2 to 7
*Sleep*	1 = less/more; 0 = 7–8 h
*Drinking*	1 = yes; 0 = no
*Smoking*	1 = yes; 0 = no
*Exercise*	1 = no; 0 = yes

### Measure

Widowhood status was measured through the question ‘What is your current marital status?’ with response options being ‘married’, ‘divorced’, ‘widowed’, and ‘never married’. We only included study respondents who had either been married or had been widowed to decrease the impact of divorce, re-marriage, or never having been married on the outcome variables of interest. We divided the respondents into two groups based on marital status collected in the CLHLS: those who are currently married and had not gone through a divorce and widowers who had not remarried (reference group) [27].

Cognitive health was assessed using the Chinese version of the Mini-Mental State Examination (MMSE) from 1995. In our study, we used education status-specific categories to define cognitive impairment based on the latest normative and validation study of the MMSE (2013) in the Chinese population, with possible scores ranging from 0 to 30 [39]. Participants with an MMSE score of ≤16 and no formal education, MMSE scores of 17–19 with 1–6 years of education, and MMSE scores of 20–23 with more than 6 years of education were categorised as having impaired cognitive function and coded as 0. Those with MMSE scores above these thresholds were classified as cognitively healthy and coded as 1.

We further used three risk factor categories as mediating variables in our model based on prior research on widowhood in older adult populations (**Table 1**): social engagement; mental state, including subjective well-being; and health behaviours, including drinking, smoking, daily exercise habits, sleep duration, and dietary behaviours.

We calculated the level of social engagement based on social activities in the last month, including visiting neighbours/friends, attending a dance class, exercising in a park or other places such as a sports or social club, etc. As most of the respondents did not engage in any social activity, we dichotomised the variable as participating in more than one activity (coded as 1) and not having any social activities (coded as 0). Older adults who had never participated in any social or outdoor activities were reported or documented as socially unhealthy.

Based on the previous studies of dietary behaviours among Chinese older adults, we selected four items from the CLHLS questionnaire [11] and recorded each its frequency by rank. The ranking levels for fruits and vegetables were ‘4 = almost every day’, ‘3 = quite often’, ‘2 = occasionally’, and ‘1 = rarely or never’, while the levels of consumption of meat and eggs were ‘3 = almost every day’, ‘2 = occasionally’, or ‘1 = rarely or never’, with combined scores ranging from 2 to 7.

Information concerning sleep duration obtained through the question: ‘How many hours on average do you sleep every day, including napping?’, whereby seven to eight hours of sleep indicated sufficient sleep duration. This classification was based on the profile study by Hirshkowitz and colleagues [40] which suggested it as the recommended amount of sleep for older adults. This recommendation was later adopted as the sleep duration guideline by the Centers for Disease Control and Prevention. We coded all sleep-related variables as binary variables.

The participants were asked whether they currently drink alcohol and/or smoke, with possible response options being ‘yes’ or ‘no’. Information on exercise habit was measured in the CLHLS using two questions: ‘Do you exercise regularly now?’ (referring to intentional fitness activities like Qigong, walking, playing ball, running, etc.) and ‘Did you previously engage in regular exercise?’ Participants who answered ‘yes’ to both questions were classified as having an exercise habit and coded as 1, and defined as ‘exercise’.

Subjective well-being in the CLHLS was measured using five items identified from prior research [41]. The questions used to measure experienced well-being were ‘Do you always look on the bright side of things?’, ‘Are you happy now as when you were younger?’, ‘Do you often feel fearful or anxious?’, ‘Do you often feel lonely and isolated?’, and ‘Do you feel the older you get the more useless you are?’. The responses ranged from 1 to 5., with ‘1’ representing ‘always or very good’ and ‘5′ representing ‘never or very bad’. We recoded these variable as 1 = the weakest feeling and 5 = the strongest feeling. This generated two indices, whereby summing items 3–5 produced a positive well-being index and summing items 1–2 gave a produced well-being index. The overall measure of well-being ranged from 5 to 25, with higher numbers indicating better well-being.

The CLHLS had ‘unable to answer’ as a response option for in many questions, which we then treated as a missing value in our analyses.

### Statistical analysis

We used descriptive statistics to summarise the general characteristics of our sample, presenting continuous variables through means/standard deviations and categorical variables through numbers/frequencies.

We first evaluated the effect of an independent variable on a dependent variable while excluding any mediating variables by using logistic regression models to analyse the effect of widowhood on cognitive health among older adults. We then conducted further analyses by including those variables that were significantly associated with the dependent variable (*P* < 0.05) and those of interest to this study in a multivariate logistic regression model. Specifically, we added social engagement, health behaviours, and subjective well-being to the model as control variables respectively to analyse various influencing factors. We reported our findings through regression coefficients and 95% confidence intervals (CIs).

We then designed a mediation analysis to determine if health behaviour and subjective well-being had a mediating role in the association between widowhood and cognitive and social health status. We evaluated the model per the following indicators: χ^2^ means and degrees of freedom (df), with χ^2^ means/df <3 indicating an acceptable fit; the comparative fit index (CFI): the Tucker-Lewis index (TLI); the standardised root means square residual (SRMR), and the root mean square error of approximation (RMSEA), with an RMSEA value ≤0.05 indicating a good model fit. The acceptable value for the non-formed fit index (NFI), goodness-of-fit index (GFI), and adjusted goodness-of-fit index (AGFI) is 0.8–0.99.

We used IBM SPSS Statistics, version 26 (IBM, SPSS Inc., NY, USA) and SPSS AMOS, version 24.0 (IBM, SPSS Inc., NY, USA) for all data analyses.

## RESULTS

### Descriptive statistics

In total, 53.8% of study participants were female; 8.8% of older adults reported cognitive impairment, while 53% reported losing a spouse (**Table 2**).

**Table 2:** Characteristics of the study sample and widowhood prevalence among Chinese older adults (n = 7796)*

Variable	n (%)	Widowhood, n (%)	χ^2^/t	*P*-value
Hukou			23.474	<0.001
*Urban*	2473 (31.7)	1212 (49.0)		
*Rural*	5323 (68.3)	2922 (54.9)		
Sex			614.479	<0.001
*Male*	3605 (46.2)	1367 (37.9)		
*Female*	4191 (53.8)	2767 (66.0)		
Age			2092.501	<0.001
*65–79*	3216 (41.3)	789 (24.5)		
*80–94*	3099 (39.8)	1986 (64.1)		
*>95*	1481 (19.0)	1359 (91.8)		
Ethnicity			0.931	0.335
*Han*	7347 (94.2)	3886 (55.2)		
*Minority*	449 (5.8)	248 (52.9)		
Year of schooling			799.600	<0.001
*0*	3395 (43.5)	2374 (69.9)		
*1–6*	2737 (35.1)	1263 (46.1)		
*>7*	1664 (21.3)	497 (29.9)		
Self-report economic status			2.045	0.727
*Very rich*	209 (2.7)	113 (54.1)		
*Rich*	1306 (16.8)	675 (51.7)		
*Average*	5527 (70.9)	2934 (53.1))		
*Poorer*	668 (8.6)	363 (54.3)		
*Very poor*	86 (1.1)	49 (57.0)		
Living status			1091.499	<0.001
*Co-habitation*	6364 (81.6)	2812 (44.2)		
*Living alone*	1221 (15.7)	1141 (93.4)		
*In nursing home*	211 (2.7)	181 (85.8)		
Smoking or not (no = 0)			135.905	<0.001
*Yes*	1245 (16.0)	472 (37.9)		
*No*	6551 (84.0)	3662 (55.9)		
Drinking or no (no = 0)			104.532	<0.001
*Yes*	1230 (15.8)	488 (39.7)		
*No*	6566 (84.2)	3646 (55.5)		
Recommended sleep duration (no = 0)			48.669	<0.001
*Yes*	2915 (37.4)	1397 (47.9)		
*No*	4881 (62.6)	2737 (56.1)		
Daily exercise or not			128.019	<0.001
*Yes*	2877 (36.9)	1285 (44.7)		
*No*	4919 (63.1)	2849 (57.9)		
Cognitive health			291.807	<0.001
*No cognitive impairment*	7113 (91.2)	3559 (50.0)		
*Impaired cognitive*	683 (8.8)	575 (84.2)		
Social health			266.076	<0.001
High social engagement	5624 (72.1)	2660 (47.3)		
Low social engagement	2172 (27.9)	1474 (67.9)		
Dietary behaviour, x̄ (SD)	4.530 (1.423)		10.399	<0.001
Subjective well-being, x̄ (SD)	16.64 (3.552)		10.893	<0.001

SD – standard deviation, x̄ – mean

*Presented as n (%) unless specified otherwise.

### Multi-level logistic regression results

Widows and widowers (84.2%) had a higher risk of having cognitive impairment than married older adults (37.9%). The results for the full model showed that being widowed was significantly associated with cognitive impairment, with the odds ratio of widows and widowers for having impaired cognition being 1.729 (95% CI = 1.312, 2.279) times higher than those who are married. Conversely, lower subjective well-being (odds ratio (OR) = 0.835; 95% CI = 0.818, 0.853), impaired social engagement (OR = 2.431; 95% CI = 1.993, 2.966), lack of daily exercise (OR = 1.585; 95% CI = 1.223, 2.053), and unhealthy dietary behaviours (OR = 0.812; 95% CI = 0.737, 0.895) were significantly associated with cognitive impairment (**Table 3**).

**Table 3:** Logistic regression analyses on cognitive impairment of older adult (n = 7796)*

Variables	Model 1	*P*-value	Model 2	*P*-value	Model 3	*P*-value	Model 4	*P*-value	Model 5	*P*-value
Hukou (ref: rural)	0.774 (0.628, 0.954)	<0.05	0.962 (0.774, 1.195)	0.726	0.811 (0.648, 1.016)	0.069	0.736 (0.594, 0.912)	<0.01	0.877 (0.693, 1.109)	0.272
Sex (ref: women)	0.901 (0.738, 1.100)	0.307	0.950 (0.758, 1.192)	0.659	0.954 (0.772, 1.179)	0.662	0.940 (0.767, 1.153)	0.553	1.007 (0.789, 1.284)	0.956
Age 80–94 (ref: 65–79)	3.987 (2.842, 5.592)	<0.001	3.733 (1.658, 5.242)	<0.001	3.727 (2.642, 5.259)	<0.001	3.373 (2.397, 4.746)	<0.001	3.162 (2.230, 4.484)	<0.001
Age >95 (ref: 65–79)	16.704 (11.765, 23.715)	<0.001	14.224 (9.992, 20.249)	<0.001	13.650 (9.519, 19.573)	<0.001	11.363 (7.944, 16.255)	<0.001	9.465 (6.534, 13.710)	<0.001
1–6 y of schooling (ref: 0 y)	0.700 (0.559, 0.877)	<0.01	0.776 (0.609, 0.964)	<0.05.	0.816 (0.641, 1.037)	0.097	0.728 (0.579, 0.916)	<0.01	0.855 (0.668, 1.094)	0.212
>7 y of schooling (ref: 0 y)	1.031 (0.761, 1.397)	0.845	1.284 (0.939, 1.755)	0.118	1.400 (1.017, 1.928)	<0.05	1.152 (0.844, 1.571)	0.372	1.664 (1.196, 2.314)	<0.01
Living alone (ref: cohabitation)	0.594 (0.459, 0.768)	<0.001	0.549 (0.422, 0.713)	<0.001	0.627 (0.478, 0.823)	<0.01	0.680 (0.523, 0.884)	<0.01	0.653 (0.494 0.863)	<0.01
In nursing home (ref: cohabitation)	2.083 (1.404, 3.091)	<0.001	2.388 (1.592, 3.581)	<0.001	2.195 (1.460, 3.300)	<0.001	2.203 (1.473, 3.295)	<0.001	2.424 (1.590, 3.697)	<0.001
Widowed or not (ref: no)	1.772 (1.369, 2.293)	<0.001	1.772 (1.366, 2.299)	<0.001	1.781 (1.358, 2.334)	<0.001	1.689 (1.299, 2.195)	<0.001	1.729 (1.312, 2.279)	<0.001
Smoking or not (ref: no)			1.116 (0.884, 1.409)	0.356					1.073 (0.836, 1.375)	0.581
Drinking or not (ref: no)			0.855 (0.678, 1.076)	0.182					0.882 (0.688, 1.131)	0.322
Daily exercise or not (ref: yes)			2.403 (1.893, 3.051)	<0.001					1.585 (1.223, 2.053)	<0.001
Recommended sleep duration (ref: yes)			1.183 (0.979, 1.429)	0.082					0.986 (0.806, 1.206)	0.887
Dietary behaviours			0.717 (0.655, 0.784)	<0.001					0.812 (0.737, 0.895)	<0.001
Subjective well-being					0.819 (0.803, 0.837)	<0.001			0.835 (0.818, 0.853)	<0.001
Social engagement (ref: healthy)							3.163 (2.639, 3.791)	<0.001	2.431 (1.993, 2.966)	<0.001
Fixed parameter	0.017	<0.001	0.039	<0.001	0.358	<0.001	0.012	<0.001	0.413	<0.05

CI – confidence interval, OR – odds ratio, ref – reference

*Presented as OR (95% CI) unless specified otherwise.

Widowed individuals who reported low social engagement were 1.182 (95% CI = 1.024, 1.365) times higher than those who are married to those who have cognitive impairment in the full model. Subjective well-being (OR = 0.961; 95% CI = 0.947, 0.976), daily exercise OR = 3.524 (95% CI = 3.074, 4.040), smoking (OR = 1.196; 95% CI = 1.031, 1.148), dietary behaviours (OR = 0.831; 95% CI = 0.782, 0.883) all showed a statistically significant association with social engagement (**Table 4**).

**Table 4:** Logistic regression analyses on social engagement of older adult (n = 7796)

Variables	Model 1	*P*-value	Model 2	*P*-value	Model 3	*P*-value	Model 4	*P*-value
Hukou (ref: rural)	1.032 (0.908, 1.173)	0.627	1.397 (1.217, 1.603)	<0.001	1.059 (0.931, 1.204)	0.384	1.399 (1.218, 1.607)	<0.001
Sex (ref: women)	0.965 (0.855, 1.089)	0.566	0.935 (0.809, 1.081)	0.366	0.982 (0.869, 1.109)	0.767	0.941 (0.814, 1.089)	0.417
Age 80–94 (ref: 65–79)	2.147 (1.865, 2.473)	<0.001	2.070 (1.790, 2.393)	<0.001	2.127 (1.865, 2.450)	<0.001	2.061 (1.782, 2.384)	<0.001
Age >95 (ref: 65–79)	5.876 (4.945, 6.981)	<0.001	5.017 (4.196, 5.997)	<0.001	5.486 (4.611, 6.527)	<0.001	4.825 (4.033, 5.774)	<0.001
1–6 y of schooling (ref: 0 y)	0.794 (0.697, 0.905)	<0.001	0.895 (0.782, 1.026)	0.111	0.823 (0.721, 0.939)	<0.01	0.911 (0.795, 1.044)	0.182
>7 y of schooling (ref: 0 y)	0.585 (0.487, 0.703)	<0.001	0.734 (0.606, 0.891)	<0.01	0.621 (0.516, 0.747)	<0.001	0.751 (0.619, 0.911)	<0.01
Living alone (ref: co-habitation)	0.619 (0.528, 0.725)	<0.001	0.590 (0.501, 0.696)	<0.001	0.627 (0.535, 0.736)	<0.001	0.598 (0.508, 0.706)	<0.001
In nursing home (ref: co-habitation)	1.201 (0.884, 1.632)	0.241	1.336 (0.967, 1.846)	0.079	1.180 (0.867, 1.604)	0.292	1.312 (0.950, 1.814)	0.100
Widowed or not (ref: no)	1.189 (1.035, 1.366)	<0.05	1.188 (1.029, 1.371)	<0.05	1.180 (1.027, 1.357)	<0.05	1.182 (1.024, 1.365)	<0.05
Smoking or not (ref: 0)			1.196 (1.032, 1.386)	<0.05			1.196 (1.031, 1.386)	<0.05
Drinking or not (ref: 0)			0.988 (0.857, 1.138)	0.863			0.996 (0.864, 1.148)	0.957
Daily exercise or not (ref: yes)			3.633 (3.171, 4.163)	<0.001			3.524 (3.074, 4.040)	<0.001
Recommended sleep duration (ref: yes)			1.207 (1.075, 1.357)	<0.01			1.181 (1.051, 1.328)	<0.01
Dietary behaviours			0.815 (0.767, 0.866)	<0.001			0.831 (0.782, 0.883)	<0.001
Subjective well-being					0.940 (0.926, 0.954)	<0.001	0.961 (0.947, 0.976)	<0.001
Fixed parameter	0.215	<0.001	0.184	<0.001	0.591	<0.001	0.329	<0.001

CI – confidence interval, OR – odds ratio, ref – reference

*Presented as OR (95% CI) unless specified otherwise.

### Mediating effect

The test of the hypothesised path model of cognitive and social engagement based on the total sample showed that it provided a good fit to the observed data (χ2 = 24.909; *P* = 0.003; χ^2^ mean/df = 2.768; GFI = 0.999; NFI = 0.995; incremental fit index (IFI) = 0.997; TLI = 0.986; CFI = 0.996; RMSEA = 0.015; root means square residual (RMR = 0.007), SRMR = 0.010.)

**Figure 1:**
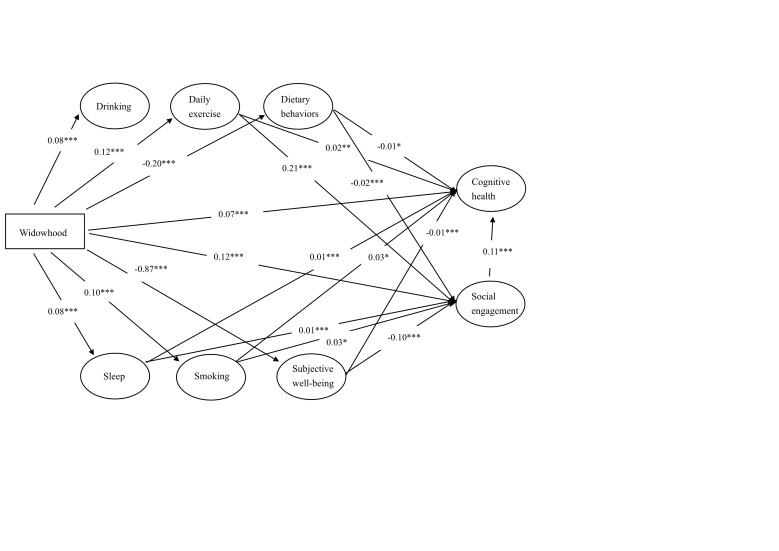
Standardised solutions for the structural model of widowhood effects on the cognitive impairment of Chinese older adults. (n = 7796). The control variables were included in the analysis but are not presented for clarity. Model fit indexes: χ2 = 24.909; *P* = 0.003; CMIN/DF = 2.768; CFI = 0.996; GFI = 0.999; NFI = 0.995; IFI = 0.997; TLI = 0.986; CFI = 0.996; TLI = 0.986; RMSEA = 0.015; RMR = 0.007; SRMR = 0.010. *P < 0.05. †*P* < 0.01. ‡*P* < 0.001.

Widowhood had a significant direct effect on the cognitive impairment of older adults (β = 0.118; *P* < 0.01), with the effect being partially mediated by social engagement, lifestyle behaviours, and subjective well-being (β = 0.075; *P* < 0.010). Compared to their married counterparts, widowed participants were less likely to engage in daily exercise (β = 0.028; *P* < 0.01) and social activities (β = 0.180; *P* < 0.01). Widowhood also had a significant direct effect on the social engagement of older adults (β = 0.132; *P* < 0.01), which was partially mediated by lifestyle behaviours and subjective well-being (β = 0.053; *P* < 0.01). Overall, the modified model explained 17.8% of the variance in cognitive health and 11.4% of the variance in social engagement (**Figure 1**, **Table 5**).

**Table 5:** Path analysis findings: total, direct, and indirect effects (n = 7796)*

Variables	Direct effect	*P*-value	Indirect effect	*P*-value	Total effect	*P*-value	SMC
Cognitive health							
*Daily exercise*	0.028	<0.01	0.041	<0.01	0.069	<0.01	
*Dietary behaviours*	−0.021		−0.009	<0.05	−0.030	<0.05	
*Subjective well-being*	−0.294	<0.05	−0.017	<0.05	−0.311	<0.05	
*Recommended sleep duration*			0.008	<0.01	0.008	<0.01	
*Smoking*			0.005	<0.05	0.005	<0.05	
*Drinking*			−0.001		−0.001		
*Social engagement*	0.180	<0.01			0.180	<0.01	0.178
*Widowhood*	0.118	<0.01	0.075	<0.01	0.193	<0.01	
Social engagement							
Daily exercise	0.229	<0.01			0.229	<0.01	
*Dietary behaviours*	−0.048	<0.05			−0.048	<0.05	
*Subjective well-being*	−0.095	<0.01			−0.095	<0.01	
*Recommended sleep duration*	0.043	<0.01			0.043	<0.01	
*Smoking*	0.026	<0.05			0.026	<0.05	
*Drinking*	−0.003				−0.003		0.114
*Widowhood*	0.132	<0.01	0.053	<0.01	0.185	<0.01	

SMC – squared multiple correlation

*Presented as β unless specified otherwise.

## DISCUSSION

Based on data from the 2018 wave of the CLHLS, we observed that widowed older adult individuals are at a higher risk of experiencing cognitive impairment than non-widowed participants. This finding is in line with the broader existing literature [42,43]. To explore the mechanisms underlying the relationship between widowhood and cognitive function, we used the stress and marital resource models, which had been well-established through prior research [12,44]. Widows and widowers often experience multiple stressors, including relocation (e.g. many of them are asked to move from home and live with their children or relatives in post-widowhood), disruptions to daily routines, increased financial strain, and the loss of a confidant [45,46]. These stressors, along with the loss of marital resources, frequently increase the risk of depression and other chronic health issues, which can detrimentally impact cognitive function [22]. However, a recent cross-sectional study of 29 242 older adult women showed that the negative relationship of widowhood to cognitive health may gradually stabilise over a period of up to 20 years [47]. Future studies could explore how the effects of widowhood on cognitive function change over time and through the widowed individual’s lifespan.

We also further explored how lifestyle behaviours alter cognitive impairment during widowhood in the context of China, a country whose traditional ethical values and cultural norms differ from those of Western societies. We specifically focussed on the relationship between social behaviour change and cognitive impairment in the widowed group. The results of our mediation model suggest that social engagement, health behaviours, and subjective well-being may play an important role in the relationship between widowhood and cognitive impairment.

We further found that social engagement is a key mediator in how widowhood affects the cognitive health of older persons. Although prior studies did not find that social engagement attributed much to the negative impacts of widowhood on cognitive outcomes [48,49], others have suggested that widowed individuals are less likely to participate in social activities [48,50]. Moreover, social disengagement in later life may result in cognitive decline [51]. The theory of socioemotional selectivity, for example, suggests may explain how spousal loss influences social engagement. Older adults focus on fewer, but more intimate relationships to satisfy their emotional needs, which helps them retain social network stability and support [52]. However, mental stress and depression after widowhood may make widowed individuals less inclined to engage in social activities [48], which means less access to social support, social integration, and social control affecting the individual’s active participation in a specific health behaviour. These factors may damage health and well-being. For instance, lower social participation not only makes the older adult lose resources in maintaining cognitive ability [36], but also leads to a high risk of cognitive impairment. This can also create a loss of protective health behaviours stemming from social participation [38]. Therefore, families should help older adults participate in activities and support them in their integration into society, either consistently or on a regular/daily basis. Society and governments can also identify the heterogeneous personal characteristics of older adults, such as social engagement traits and marital situations, and use them to formulate effective, targeted interventions to minimise the loss of social resources.

Our results suggest that a portion of the connection between widowhood and cognitive impairment could be attributed to health behaviours. Earlier studies have shown that widowed individuals were more likely to smoke than their non-widowed counterparts [16]. Reduced sleep duration and irregular healthy food habits likewise emerged as negative effects of widowhood [15,16]. This is also consistent with the theory of socioemotional selectivity, which posits that increased stress from marital dissolutions and the absence of partner support can result in detrimental adjustments and emotional or behavioural issues, thereby potentially harming cognitive function and hastening cognitive decline in later life [53].

We also observed a decrease in physical daily exercise and an increased rate of drinking following the death of a spouse. Prior studies have not determined if or how much physical activity varies after becoming widowed, with divergent results reporting increases [14,16], decreases [15,54], or no changes [55] in physical activity during the transition to widowhood. In a four-year study of 80 944 women aged 46–71, those who became widowed and remained unmarried decreased their intake of alcohol 0.43 servings/week during the aftermath of the loss of their spouse [16]. Another study of 38 865 men aged 40–75 found an association between marital dissolution and increased alcohol consumption [15]. The benefits of marriage are known to affect men and women differently. The marital role of women is more likely to be characterised by caregiving, which may indicate that women do more housework and less physical exercise [56]. Additionally, due to traditional gender roles, women consume less alcohol overall [16]. Therefore, future research should explore the effect of gender and marital roles in this context.

Finally, we determined subjective well-being to be an essential mediating variable in the association between widowhood and cognitive impairment. The loss of a spouse tends to reduce social networking and harm social connections, with widowed older adults receiving less social support and spiritual comfort, negatively impacting their life satisfaction [57]. High levels of subjective well-being are considered a key factor in positive ageing [58]; it has also been found to have a strong relationship with specific cognitive functions [59]. However, few previous studies have tested this mediating effect of subjective well-being. In the context of global population ageing, the mental health of older people has emerged as a topic of societal importance and concern. As subjective well-being seemingly plays a mediating role in the pathway between losing a loved one and these damaging cognitive health effects, targeted interventions and preventive measures could help offset negative outcomes in the future.

Our study has several limitations, emphasizing the need for additional research to fully understand the relationship between widowhood and health. First, we used a cross-sectional design, preventing us from establishing causal inferences between widowhood and cognitive impairment. Despite this, our findings are based on a nationally representative sample, providing relevant data for health promotion among widowed older adults. Second, we did not account for factors like marriage quality and widowhood duration in our analysis, although they have been found to harm the cognitive function of elderly individuals [60]. We were also unable to determine the impact of changes in lifestyle behaviours and social participation, especially if they progressively diminish over time following the loss of a spouse. Third, previous investigations have explored that widowhood might potentially increase the mortality of elderly individuals. Our sample also did not include people who died due to health problems brought on by he loss of a spouse, which caused the survivor bias in our present samples leading to an underestimate of the detrimental impact between widowhood and cognitive function.

## CONCLUSIONS

By analysing data of the 2018 wave of the CLHLS, we explored the mediating role of social engagement, health behaviours, and subjective well-being in the association between widowhood and cognitive impairment. We found that widowed older adults had a higher risk of having cognitive impairment than their married counterparts, and that the effect of widowhood on cognitive impairment was partially mediated by social engagement, lifestyle behaviours, and subjective well-being. Our findings highlight a need for policies that focus on the specific requirements of this vulnerable population, such as the maintenance of social interaction, adoption of a healthy lifestyle, improvement of subjective well-being, and provision of necessary support systems.
